# Rice and Bean Targets for Biofortification Combined with High Carotenoid Content Crops Regulate Transcriptional Mechanisms Increasing Iron Bioavailability

**DOI:** 10.3390/nu7115488

**Published:** 2015-11-23

**Authors:** Desirrê Morais Dias, Maria Eliza de Castro Moreira, Mariana Juste Contin Gomes, Renata Celi Lopes Toledo, Marilia Regini Nutti, Helena Maria Pinheiro Sant’Ana, Hércia Stampini Duarte Martino

**Affiliations:** 1 Department of Nutrition and Health, Federal University of Viçosa, Viçosa 36570-000, Minas Gerais, Brazil; elizamoreira@yahoo.com.br (M.E.C.M.); mariana.contin@ufv.br (M.J.C.G.); renatacelly@yahoo.com.br (R.C.L.T.); helena.santana@ufv.br (H.M.P.S.A.); hercia72@gmail.com (H.S.D.M.); 2 EMBRAPA Food Technology, Rio de Janeiro 23020-470, Brazil; marilia.nutti@embrapa.br

**Keywords:** gene expression, antioxidant capacity, iron, sweet potato, pumpkin, biofortification

## Abstract

Iron deficiency affects thousands of people worldwide. Biofortification of staple food crops aims to support the reduction of this deficiency. This study evaluates the effect of combinations of common beans and rice, targets for biofortification, with high carotenoid content crops on the iron bioavailability, protein gene expression, and antioxidant effect. Iron bioavailability was measured by the depletion/repletion method. Seven groups were tested (*n* = 7): Pontal bean (PB); rice + Pontal bean (R + BP); Pontal bean + sweet potato (PB + SP); Pontal bean + pumpkin (PB + P); Pontal bean + rice + sweet potato (PB + R + P); Pontal bean + rice + sweet potato (PB + R + SP); positive control (Ferrous Sulfate). The evaluations included: hemoglobin gain, hemoglobin regeneration efficiency (HRE), gene expression of divalente metal transporter 1 (DMT-1), duodenal citocromo B (DcytB), ferroportin, hephaestin, transferrin and ferritin and total plasma antioxidant capacity (TAC). The test groups, except the PB, showed higher HRE (*p* < 0.05) than the control. Gene expression of DMT-1, DcytB and ferroportin increased (*p* < 0.05) in the groups fed with high content carotenoid crops (sweet potato or pumpkin). The PB group presented lower (*p* < 0.05) TAC than the other groups. The combination of rice and common beans, and those with high carotenoid content crops increased protein gene expression, increasing the iron bioavailability and antioxidant capacity.

## 1. Introduction

The deficiency of micronutrients, collectively known as hidden hunger, affects approximately two billion people worldwide. Iron (Fe) deficiency is the most prevalent nutrient deficiency, affecting around 40% of the world population, particularly women and children in developing countries [1,2].

Global efforts to reduce the incidence of this nutritional deficiency have been directed to increase the consumption of micronutrient through fortification and biofortification of food. The biofortification of staple crops is a new public health approach to control deficiency of vitamin A, iron and zinc in poor countries. The biofortification process produces foods with higher micronutrient concentrations through the best practices of breeding and modern biotechnology [3,4].

However, there is a need to assess the effect of interactions between biofortified foods consumed in the same diet [5], since the concomitant intake of foods influences the bioavailability of nutrients. Moreover, increased consumption of iron [6] and vitamins can influence the content and activity of antioxidant enzymes [7,8].

Iron fortification is often ineffective due to concomitant deficiency of vitamin A; the consumption of this vitamin has a close relationship with iron status, as shown in studies with animals and humans with iron deficiency anemia [9,10,11,12].

Furthermore, the increase in iron concentration in food crops may not result in proportional increment of absorbed iron, as crop varieties with high iron content can also have increased or decreased concentrations of inhibitors or enhancers of iron absorption. Therefore, there is a need to analyze the concentration of iron and its bioavailability in new cultures with high concentrations of minerals and carotenoids [13]. In addition, the body’s need for iron is the most important factor that guides iron absorption and this homeostasis is regulated by transcriptional mechanisms, through regulation of gene expression of proteins involved in iron metabolism [14].

Some studies have assessed the availability of iron in biofortified crops such as common beans [15,16,17] and iron bioavailability of iron fortified rice [18,19]. However, there are no studies about iron bioavailability of combinations of food in the micronutrient biofortification program. Moreover, the biofortification program with iron has not been successful in increasing the bioavailability of iron to desired levels, which may impact the nutritional status in animals and in humans [20]. The association with vitamin A sources can point to an alternative for improving the effectiveness of biofortification on human health. Thus, the objective of this study was to evaluate the effect of food combinations of beans and rice, targets for biofortification, with high carotenoid content crops (sweet potato and pumpkin) on the bioavailability of iron, gene expression of proteins involved in iron metabolism, and their antioxidant effect.

## 2. Experimental Section

### 2.1. Sample

Staple food crops from Brazilian Biofortification Program were evaluated: common beans (*Phaseolus vulgaris* L.) BRS Pontal (high Fe content); white rice (*Oryza sativa*) Chorinho (source of zinc); pumpkin (*Curcubita moscata*) Duchesne and sweet potato (*Ipomoea batatas*) (high pro-vitamin A carotenoid content). Cultivars were developed and supplied by Empresa Brasileira de Pesquisa Agropecuária (EMBRAPA, Brazil).

Briefly, the biofortification crop improvement was divided into three phases: (1) Early-stage product development and parent building; (2) Intermediate product development; (3) Final product development. BRS Pontal is a promising cultivar for biofortification, since it does not present the 100% of target for biofortification, but it has the minimum amount of iron required to be considered as an intermediate product development (75 µg·Fe/g). BRS Pontal has 50% of the target (50 ppm + 25 ppm iron), while the pumpkin and sweet potato have 100% of the target levels of 30 µg/g of pro-vitamin A carotenoids.

### 2.2. Staple Food Crop Flours Preparation

The common beans were cooked in three replicates in a conventional pressure cooker for 40 min using a bean/water ratio of 1:2.2 (*w*/*v*) and dried in an air oven for 17 h at 60 °C [21]. The rice was cooked in three replicates in a conventional cooker using a rice/water ratio of 1:2.83 (*w*/*v*) and dried in an air oven (Nova Ética, São Paulo, Brazil) for 17 h at 60 °C. The pumpkin and sweet potato were peeled and sliced on a multiprocessor (Philips Walita, Amsterdam, The Netherland) and dried in an air oven for 6 hours at 60 °C [22]. All foods were ground by mill 090 CFT (Marconi, São Paulo, Brazil) at 3000 rpm, sieved (600 mesh screen) and stored at −12 °C.

### 2.3. Determination of Iron and Zinc 

The determination of iron and zinc content of food and the iron content of the diet were performed according to Gomes (1996) [23]. Briefly, 1.0 g of the samples was oxidized with 10 mL of nitric acid for 8 h at room temperature. Later, the samples were heated in the digester block with exhaust to approximately 120 °C for 16 h. The iron and zinc concentrations were determined by coupled plasma atomic emission spectrometry (model Optima 3300 DV, Perkin Elmer, MA, USA), with an inducible plasma argon source. Analysis was performed under the following conditions: power of 1300 W, plasma argon flow rate of 15 L·min^−1^, auxiliary argon flow rate of 0.7 L·min^−1^, nebulizer argon flow rate of 0.5 L·min^−1^, rate of sample introduction of 1.5 mL·min^−1^. Calibration curves were used to prepare standard solutions of iron and zinc concentration, according to Pires *et al.*, 2015 [24]. The analyses were performed in triplicate (Table 1).

**Table 1:** Chemical composition and phytate/iron and zinc/iron molar ratio of flours food inserted in the biofortification program *, on dry basis.

	Pontal Bean	Rice	Pumpkin	Sweet Potato
Moisture (g·100 g^−1^)	10.7 ^a^ ± 0.28	7.35 ^d^ ± 0.06	9.99 ^a,b^ ± 0.55	9.92 ^b^ ± 0.06
Ash (g·100 g^−1^)	3.14 ^b^ ± 0.03	0.34 ^e^ ± 0.02	6.38 ^a^ ± 0.07	2.27 ^d^ ± 0.06
Lipids (g·100 g^−1^)	1.37 ^b^ ± 0.3	0.13 ^c^ ± 0.13	1.46 ^b^ ± 0.14	1.55 ^e^ ± 0.34
Protein (g·100 g^−1^)	18.86 ^b^ ± 0.08	8.83 ^d^ ± 0.18	15.86 ^c^ ± 0.24	2.63 ^e^ ± 0.12
Total dietary fiber (g·100 g^−1^)	26.69 ^a^ ± 0.45	1.08 ^c^ ± 0.1	15.02 ^b^ ± 0.03	15.31 ^b^ ± 0.31
*Soluble fiber*	7.04 ± 1.27	0.47	5.10 ± 0.25	4.89 ± 0.63
*Insoluble fiber*	19.64 ± 0.92	0.61	9.92 ± 0.23	10.42 ± 0.38
Carbohydrates (g·100 g^−1^)	48.87 ^b,c^ ± 0.73	82.48 ^a^ ± 0.05	52.19 ^b,c^ ± 0.34	69.62 ^a,c^ ± 0.56
Total phenolic (mg·de·EqGA/g)	1.33 ^b^ ± 0.15	0.06 ^d^ ± 0.01	2.41 ^a^ ± 0.12	1.51 ^b^ ± 0.07
Carotenoids (mg/100 g)	nd	nd	308.84 ^a^ ± 1.98	127.11 ^b^ ± 0.06
**Minerals**				
*Iron (mg/100 g)*	7.52 ± 0.1	3.9 ± 0.03	2.09 ± 0.18	3.33 ± 0.06
*Zinc (mg/100 g)*	3.11 ± 0.01	1.73 ± 0.06	1.71 ± 001	1.8 ± 0.05
Phytic acid (g/100 g)	0.51 ^a^ ± 0.02	0.20 ^b^ ± 0.03	0.03 ^c^ ± 0.32	0.10 ^c^ ± 0.1
**Molar Ratio**				
*Phytate/iron*	5.78	4.38	1.26	2.54
*Zinc/iron*	0.35	0.37	0.72	0.46

Data presented as mean and standard deviation. nd: not determined. Means with different letters in the same line present significant difference (*p* < 0.05) by Tukey test. * BIOFORT/HarvestPlus.

### 2.4. Determination of Carotenoids

The concentration of pro-vitamin A carotenoids (α and β-carotene) in pumpkin and sweet potato was determined by Rodriguez *et al.* (1976) [25]. Five grams of sample were ground in 60 mL of chilled acetone for approximately 2 min and the material was vacuum filtered on a Buchner funnel using filter paper. The filtrate was transferred to a separator funnel, in which 50 mL of cooled petroleum ether were added to transfer the pigment to the acetone ether. Each fraction was washed three times with distilled water to remove all acetone. The concentration of material was performed by evaporation of the petroleum ether extract using a rotary evaporator at 35 °C. The pigments were re-dissolved in a known amount of petroleum ether and stored in amber glass vials at −18 °C.

For analysis, an aliquot (2 mL) of the extract stored in petroleum ether was evaporated under nitrogen flow and then recovered in the same amount of methanol and filtered through a filter unit with 0.45 µM porosity. The analyses of carotenoids were performed in triplicate by high performance liquid chromatography (HPLC) using the chromatographic conditions developed by Pinheiro-Sant’Ana *et al.* (1998) [26] as follows: HPLC-DAD system (diode array detector); chromatographic column Phenomenex Gemini RP-18, 250 × 4.6 mm, 5 μm, equipped with Phenomenex ODS guard column (C18), 4 mm × 3 mm. The mobile phase was methanol: ethyl acetate: acetonitrile (80:10:10, *v*/*v*/*v*) at a flow rate of 2.0 mL/min.

### 2.5. Phytate and Phenolic Compounds

Phytate content was determined in triplicate by ion exchange and spectrophotometry according to Latta and Eskin (1980) [27], with modifications by Ellis and Morris (1986) [28]. The determination of the concentration of phenolic compounds in foods was performed using the Folin Ciocalteu reagent as described by Singleton *et al.* (1999) [29].

### 2.6. Animals and Diets

Controlled experimental tests were used and the bioavailability of iron was evaluated by the hemoglobin depletion/repletion method modified by the Association of Official Analytical Chemistry (AOAC, 2012) [30]. At 12 days of age 49 male rats (*Ratus norvegicus albinus* Wistar) from the Central Animal Facility of the Center for Life Sciences and Health at Federal University of Viçosa, Minas Gerais, Brazil, were placed in individual temperature-controlled (22 ± 2 °C) cages, with a photoperiod of 12 h. The experimental diets were based on the standard AIN-93G [31] (Table 2) diet for rodents.

Animals initially received a depletion diet containing Fe-free mineral mixture to reduce hemoglobin (Hb) concentrations and deionized water *ad libitum*, for 21 days. Animals were then divided into seven groups (*n* = 7) such that the Hb concentration was not statistically different among groups: (1) Pontal Bean; (2) Pontal Bean and Rice; (3) Pontal Bean and Pumpkin; (4) Pontal Bean and Sweet Potato; (5) Pontal Bean + Rice + Pumpkin; (6) Pontal Bean, Rice and Sweet Potato; (7) Control (containing ferrous sulfate as iron source). The repletion diet was pair fed to control food and Fe intake, and deionized water was offered *ad libitum*, for 14 days.

During the repletion phase the staple food crops were used as source of iron, and the ferrous sulfate was used as positive control. All treatments offered 12 mg of iron per kg of diet [32]. The quantity of pumpkin and sweet potato added in the experimental diets was calculated to provide 4.5 mg of vitamin A per kg of diet (Table 3). This value was based on the conversion of milligrams of vitamin A per gram of body weight in the study by Mwanri *et al.* (2000) [33] in which anemic children between 9 and 12 years old were supplemented with 1.5 mg of vitamin A per day to assist in the recovery of iron status. At the end of depletion and repletion phases blood samples were collected from the rat tails to determine Hb concentrations.

On the 36th day, after 12 h fasting, the animals were anesthetized with isoflurane (Isoforine, Cristália^®^, Itapira, Brazil) and were euthanized by cardiac puncture. Blood and fragments of the liver and duodenum were collected.

All experimental procedures with animals were performed in accordance with the ethical principles for animal experimentation and the study approved by the Ethics Committee of the Federal University of Viçosa.

### 2.7. Blood Tests

Serum hemoglobin was measured by cyanide methemoglobin method, proposed by the AOAC (2012) [30], using a colorimetric kit for *in vitro* diagnosis. A volume of 10 mL of blood was pipetted and mixed with 5 mL of Drabkin’s solution color reagent (containing potassium cyanide, and hydrogen cyanide). The reading of absorbance was done in UV-Visible Multiskan (Thermo Scientific, Massachusetts, MA, USA) at a wavelength of 540 nm.

**Table 2:** Food and nutritional composition of experimental diets.

	Standard Diet without Iron	Standard Diet with Iron (Ferrous Sulfate)	Pontal Bean	Pontal Bean and Rice	Pontal Bean and Pumpkin	Pontal Bean and Sweet Potato	Pontal Bean, Rice and Pumpkin	Pontal Bean, Rice and Sweet Potato
***Ingredients (1 kg of Diet)***								
Ferrous Sulfate (mg)	-	120.98	-	-	-	-	-	-
Common Bean (g)	-	-	163.73	100.84	156.36	155.16	91.41	88.05
Rice (g)	-	-	-	100.84	-	-	91.41	88.05
Pumpkin (g)	-	-	-	-	25.56	-	25.56	-
Sweet Potato (g)	-	-	-	-	-	18.85	-	18.85
Albumin (g)	200.00	200.00	173.44	178.11	170.18	174.98	177.65	180.93
Dextrinized starch (g)	132.00	132.00	132.00	132.00	132.00	132.00	132.00	132.00
Sucrose (g)	100.00	100.00	100.00	100.00	100.00	100.00	100.00	100.00
Soybean Oil (mL)	70.00	70.00	67.71	68.62	67.85	67.87	69.88	69.88
Microcrystalline cellulose (g)	50.00	50.00	10.98	24.96	9.33	10.42	24.73	24.70
Mineral Mix without iron (g)	35.00	35.00	35.00	35.00	35.00	35.00	35.00	35.00
Vitamin Mix (g)	10.00	10.00	10.00	10.00	10.00	10.00	10.00	10.00
l-cystine (g)	3.00	3.00	3.00	3.00	2.73	3.00	3.00	3.00
Choline Bitartrate (g)	2.50	2.50	2.50	2.50	2.50	2.50	2.50	2.50
Corn starch (g)	397.50	397.50	337.89	309.32	333.10	337.20	305.41	302.83
***Nutritional Composition***								
Total calories (Kcal)	3830.8	3830.8	3989.47	4105.9	4013.1	4028.6	4100.4	4093.5
Caloric density (Kcal/g)	3.83	3.83	3.98	4.1	4.01	4.02	4.1	4.09
Vitamin A (mg/kg)	1.20	1.20	1.20	1.20	5.70	5.70	5.70	5.70
Iron (mg/kg)	0.30	20.4 *	23.7 ± 0.81 *	19.7 ± 0.68 *	26.3 ± 4.7 *	22.5 ± 0.09 *	23.9 ± 3.62 *	22.7 ± 0.82 *
Phytate (g/100 g)	nd	nd	0.83	0.72	0.805	0.81	0.63	0.66
Phytate: iron molar ratio	nd	nd	29.81 ^a^ ± 1.01	30.79 ^a^ ± 1.07	30.25 ^a^ ± 0.12	26.89 ^a^ ± 4.8	22.88 ^a^ ± 3.46	24.86 ^a^ ± 0.9
Zinc: iron molar ratio	-	-	0.18 ^a^ ± 0.006	0.21 ^a^ ± 0.007	0.2 ^a^ ± 0.0008	0.17 ^a^ ± 0.03	0.17 ^a^ ± 0.02	0.18 ^a^ ± 0.006

* Analyzed according to the methodology proposed by Gomes (1996) [23]; nd: not determined. Means with different letters in the same line present significant difference (*p* < 0.05) by Tukey test.

**Table 3:** Sequence of primers used in the RT-PCR analysis.

Genes	Oligonucleotide (5’–3’)	
	Forward	Reverse
**GAPDH**	AGGTTGTCTCCTGTCACTTC	CTGTTGCTGTAGCCATATTC
**DMT-1**	CTGATTTACAGTCTGGAGCAG	CACTTCAGCAAGGTGCAA
**DcytB**	TGCAGACGCAGAGTTAAGCA	CCGTGAAGTATACCGGCTCC
**Ferroportin**	TTCCGCACTTTTCGAGATGG	TACAGTCGAAGCCCAGGACCGT
**Hephaestin**	GGCACAGTTACAGGGCAGAT	AGTAACGTGGCAGTGCATCA
**Ferritin**	CAGCCGCCTTACAAGTCTCT	ATGGAGCTAACCGCGAAGAC
**Transferrin**	AGCTGCCACCTGAGAACATC	CGCACGCCCTTTATTCATGG

GAPDH: glyceraldehyde 3-phosphate dehydrogenase; DMT-1: Divalent metal transporter-1 Protein; DcytB: Duodenal cytochrome B.

### 2.8. Iron Bioavailability

The iron bioavailability was calculated according to Hernandez *et al.* (2003) [34]. The hemoglobin regeneration efficiency (ERH%) was calculated by the expression: ERH% = ((mg Fe final Hb − mg Fe initial Hb)/100)/mg Fe consumed. The iron in hemoglobin content was estimated by: (Body weight (g) × Hb (g/L) × 0.335 × 6.7)/1000. This variable was calculated assuming the total blood volume to be 6.7% of the mouse body weight, and the body iron in hemoglobin content as being 0.335. The iron utilization was calculated as: (ERH% × dietary iron)/100, and the absorption of iron was calculated as: (Fe intake − excretion Fe) Fe intake.

### 2.9. Extraction of mRNA and Expression of Proteins Involved in Iron Metabolism

The organs were macerated in liquid nitrogen in RNase free conditions and samples were aliquoted for total RNA extraction. Total RNA was extracted with Trizol reagent (Invitrogen, Carlsbad, CA, USA) using the manufacturer's recommendations. Two µL of mRNA extracted at a concentration of 200 µL^−1^ was used to synthesize the cDNA using M-MLV reverse transcription kit (Invitrogen Corp., Grand Island, NY, USA) according to the manufacturer’s protocol.

### 2.10. Determination of Gene Expression of Proteins Involved in Iron Metabolism by Reverse Transcriptase Polymerase Chain Reaction (RT-PCR)

Expression of mRNA levels in the duodenal mucosa and the liver of proteins involved in iron metabolism were analyzed by RT-PCR. The SYBR green marker and PCR master mix from Applied Biosystems (Foster City, CA, USA) were used and analyses were performed on the StepOne™ Real-Time PCR System (Thermo Fisher Scientific) using the measurement system by SYBR-Green Fluorescence and Primer Express software (Applied Biosystems, Foster City, CA, USA). The PCR involved an initial denaturation cycle of 95 °C (10 min) and then 40 cycles with 1 min denaturation (94 °C), 1 min annealing (56 °C) and 2 min elongation (72 °C), followed by a standard dissociation curve. Sense and antisense primer sequences (GenOne Biotechnologies, Rio de Janeiro, Brazil) were used to amplify proteindivalent metal carrier (DMT-1), ferroportin, hephaestin and duodenal cytochrome b (DcytB) (Sigma-Aldrich, Missouri, MO, USA), and the proteins ferritin and transferrin from liver (GenOne Biotechnologies, Rio de Janeiro, Brazil). The relative expression levels of mRNA were normalized by the endogenous control glyceraldehyde 3-phosphate dehydrogenase (GAPDH). All steps were performed using open conditions with RNase.

### 2.11. Plasma Total Antioxidant Capacity

The plasma total antioxidant capacity was measured by the colorimetric method with the Sigma kit (Sigma-Aldrich, St. Louis, MO, USA). The concentration of antioxidants in plasma was expressed as mM Trolox equivalent.

### 2.12. Statistical Analysis

The flours of staple food crops were analyzed in replicates. Chemical composition data was subjected to analysis of variance (ANOVA). The post hoc Tukey test was used to compare the groups. Experimental treatments were arranged in a completely randomized design, with seven repetitions. The results were analyzed by ANOVA. For significant “F-value”, post hoc Dunnett test was used to compare each test group to the control group. Test was performed in order to compare test groups. The mean dispersion was expressed as standard deviation. Statistical analyses were carried out using GraphPad Prism version 5.0 software (GraphPad Software, California, CA, USA). The level of significance was established at *p* < 0.05.

## 3. Results

### 3.1. The Effect of Combinations of Staple Food Crops on the Bioavailability of Iron

The groups fed with diets containing Pontal bean and high pro-vitamin A carotenoids crops (PB + P; PB + SP; PB + R + P; PB + R + P) had hemoglobin gain similar to the positive control group, which received ferrous sulfate as a source of iron (*p* ≥ 0.05) (Table 4). The animals fed with diets containing only Pontal bean had Hemoglobin Gain (HG) and Hemoglobin Maintenance Efficiency (HRE) inferior to the control and other groups (*p* < 0.05). When the Pontal bean was associated with the rice (PB + R) the HG remained lower than the control, but the HRE was similar (*p* ≥ 0.05) to the other test groups.

**Table 4:** Total intake of iron and vitamin A and indices for assessing iron bioavailability (*n* = 7).

	Fe Intake	Vitamin A Intake	HG	%HRE	RBV-HRE
FS	6.75 ^a^ ± 0.19	0.39 ^b^ ± 0.01	6.9 ^A^ ± 1.95	76.92 ^B^ ± 0.4	-
PB	5.13 ^b,c^ ± 0.28	0.39 ^b^ ± 0.02	2.6 ^B,b^ ± 1.33	60.71 ^C,b^ ± 0.15	0.79 ^b^ ± 0.2
PB + R	4.28 ^e^ ± 0.33	0.39 ^b^ ± 0.03	3.84 ^B,a^ ± 1.03	87.52^A,a^ ± 0.16	1.14 ^a^ ± 0.21
PB + P	4.6 ^d,e^ ± 0.38	1.29 ^a^ ± 0.11	4.85 ^A,a^ ± 1.16	86.75 ^A,a^ ± 0.12	1.13 ^a^ ± 0.16
PB + SP	5.37 ^b^ ± 0.67	1.28 ^a^ ± 0.16	5.77 ^A,a^ ± 2.6	86.72 ^A,a^ ± 0.24	1.13 ^a^ ± 0.32
PB + R + P	5.2 ^b,c^ ± 0.15	1.37 ^a^ ± 0.04	4.72 ^A,a^ ± 1.56	81.65 ^A,a^ ± 0.12	1.06 ^a,b^ ± 0.15
PB + R + SP	4.81 ^c,d^ ± 0.37	1.33 ^a^ ± 0.1	4.97 ^A,b^ ± 1.85	85.72 ^A,a^ ± 0.24	1.05 ^a,b^ ± 0.33

Data presented as mean and standard deviation. FS: Ferrous Sulfate; PB: Pontal Bean; PB + R: Pontal Bean + Rice; PB + P: Pontal Bean + Pumpkin; PB + SP: Pontal Bean + Sweet Potato; PB + R + P: Pontal Bean + Rice + Pumpkin; PB + R + SP: Pontal Bean + Rice + Sweet Potato. HG: Hemoglobin Gain; HRE: Hemoglobin Maintenance Efficiency; RBV: Relative Biological Value of HRE. Means followed by different capital letters in columns differ by Dunnett’s test (*p* < 0.05). Means followed by different small letters differ by Duncan test (*p* < 0.05).

### 3.2. Gene Expression of Proteins Involved in Iron Metabolism

**Figure 1:**
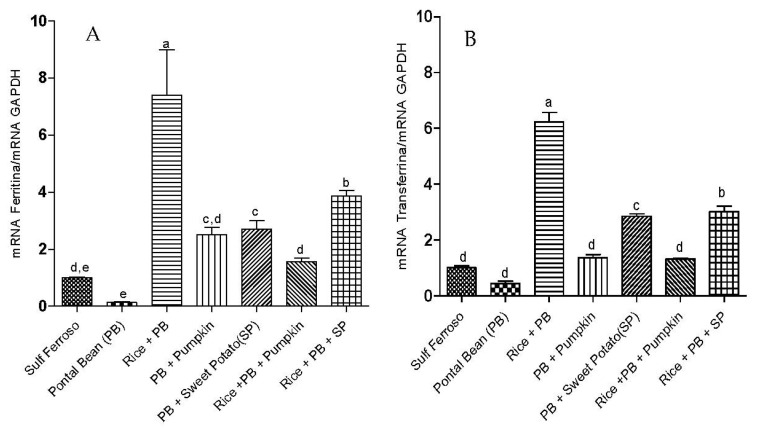
Effect of the ingestion of different combinations of staple food crops from micronutrients biofortification program and ferrous sulfate on the gene expression of proteins in liver tissue. RT-PCR Analysis. (**A**) Ferritin; (**B**) Transferrin. FS: Sulfate Ferrous; PB: Pontal Bean; PB + R: Pontal Bean + Rice; PB + A: Pontal Bean + Pumpkin; PB + SP: Pontal Bean + Sweet Potato; PB + R + P: Pontal Bean + Rice + Pumpkin; PB + R + SP: Pontal Bean + Rice + Sweet Potato. Different letters indicate statistical differences at 5% probability by Duncan test.

The PB + R group showed higher expression of ferritin (Figure 1A) and transferrin (Figure 1B) (*p* < 0.05), followed by PB + R + SP group. Furthermore, the level of expression of these two proteins was proportional in all groups.

**Figure 2:**
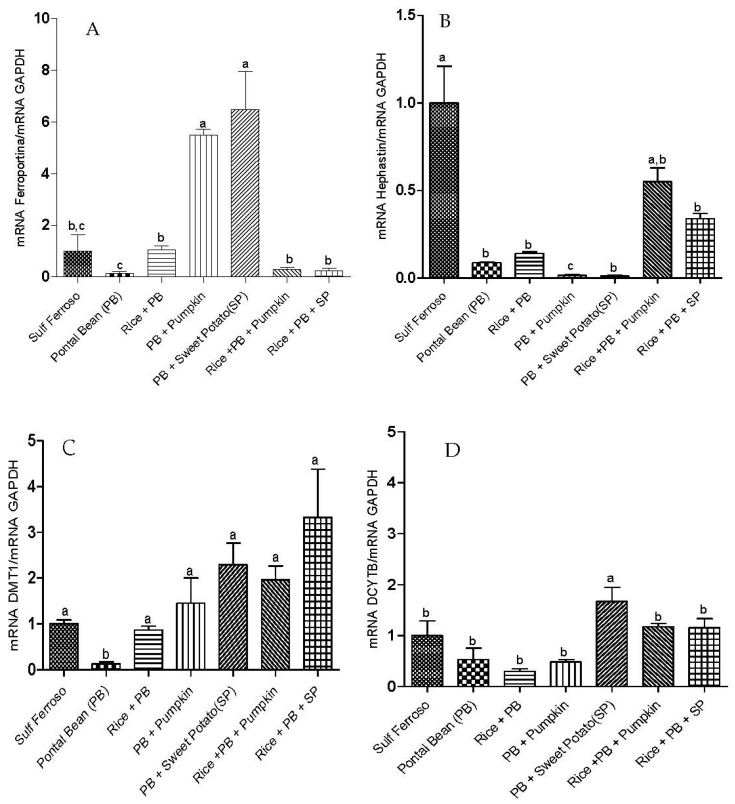
Effect of the ingestion of different combinations of staple food crops from micronutrients biofortification program and ferrous sulfate on the gene expression of proteins in duodenal tissue. RT-PCR Analysis. (**A**) Ferroportin; (**B**) Hephaestin; (**C**) DMT-1; (**D**) Dcytb. FS: Sulfate Ferrous; PB: Pontal Bean; PB + R: Pontal Bean + Rice; PB + P: Pontal Bean + Pumpkin; PB + SP: Pontal Bean + Sweet Potato; PB + R + P: Pontal Bean + Rice + Pumpkin; PB + R + SP: Pontal Bean + Rice + Sweet Potato. Different letters indicate statistical differences at 5% probability by Duncan test.

The animals fed diets containing only Pontal bean had lower gene expression of proteins involved in iron metabolism, which may explain the lower HRE (*p* < 0.05). Additionally, groups containing Pontal bean and high pro-vitamin A carotenoids crops (PB and PB + R + SP) had increased mRNA expression of DcytB (Figure 2D) and ferroportin (Figure 2A).

The hephaestin mRNA was more expressed (*p* < 0.05) in the control group and the groups receiving diets containing rice, bean and high pro-vitamin A carotenoids crops (PB + R + P and PB + R + SP) (Figure 2B).

### 3.3 The Effect of the Combinations of Staple Food Crops on Plasma Total Antioxidant Capacity

**Figure 3:**
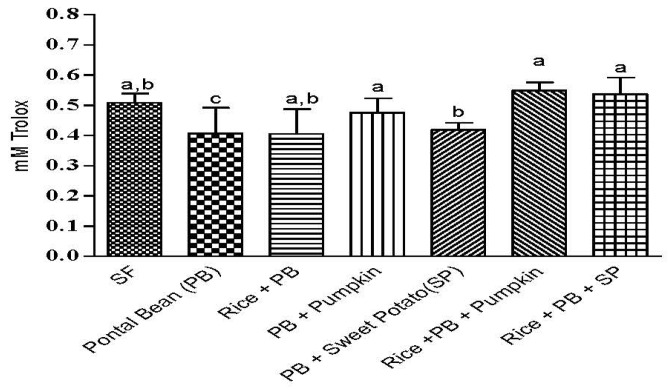
Plasma Total Antioxidant capacity. Results are expressed in mM Trolox equivalent. FS: Sulfate Ferrous; PB: Pontal Bean; PB + R: Pontal Bean + Rice; PB + P: Pontal Bean + Pumpkin; PB + SP: Pontal Bean + Sweet Potato; PB + R + P: Pontal Bean + Rice + Pumpkin; PB + R + SP: Pontal Bean + Rice + Sweet Potato. Different letters indicate statistical differences at 5% probability by Duncan test.

The group fed only Pontal bean showed lower (*p* < 0.05) antioxidant capacity (Figure 3). The other tested groups in which Pontal bean was combined with rice, pumpkin and sweet potato was similar (*p* ≥ 0.05) to the control.

## 4. Discussion

Several studies have demonstrated increased bioavailability of iron from biofortified foods such as rice and common bean [14,17,19,34,35]. However, the effect of the combination of staple crops enriched with iron or carotenoids on the bioavailability of iron had not yet been tested. The present study showed the importance of combining these foods in the diet in order to increase the iron content, enhance compounds associated with iron absorption, and to minimize the negative effect of phytochemicals.

The staple foods that are a part of the usual intake in Brazil, such as rice and common beans, combined with high pro-vitamin A carotenoid content crops increased the bioavailability of iron. Vitamin A can act in iron mobilization of organic tissue stocks, favoring the availability of this mineral for hematopoiesis and hemoglobin synthesis [36]. Additionally, vitamin A has been associated with the gene expression of hepcidin, a hormone that regulates uptake and export of endogenous iron, in the liver [37].

In the present study, the molar ratio of phytate: iron did not differ among diets and was around 27. Both *in vitro* studies and studies in humans have shown that the molar ratio of phytate: iron from 4 to 30 could significantly inhibit the absorption of iron [16,38,39,40]. The combination of Pontal bean target for biofortification with high pro-vitamin A carotenoid content crops increased the iron absorption, even in diets with a high molar ratio of phytate: iron. Rats have the intestinal phytase, but studies show that young rats, such as the animals used in this study, have low activity of this enzyme [4]. In the case that phytase had been active only in the group fed with beans they would have presented iron bioavailability similar to ferrous sulfate. Thus, the most likely case is that vitamin A bound with iron to form a complex which acts as a chelating agent preventing the inhibitory effect of phytate on the iron absorption [41,42].

The animals that received an additional dose of pro-vitamin A carotenoid with common beans (PB + SP and PB + P) showed enhanced expression of ferroportin, which also may have contributed to increase the iron efflux and incorporation of iron in the hemoglobin resulting in higher GHb values. Since retinoic acid is the main active form of vitamin A it was capable to induce the expression of ferroportin gene in Caco-2 cells through a hepcidin-independent mechanism [43].

The PB group showed lower HG than other groups and when the iron consumption was corrected by iron incorporated into hemoglobin, the PB group remained with the lowest value of HRE. The poor performance of this group may be associated with a lower mRNA expression of DMT-1 and ferroportin, proteins that are necessary for the absorption of iron and outsourcing enterocytes [44]. Some studies had showed lower gene expression of these proteins in anemic animals, in order to compensate the poor Fe status [17,45,46]. However, in our experimental design, the iron deficiency was induced in the animals, and then this deficiency was corrected by intake of tested foods. Therefore, at the end of the experiment, each animal, including the control, had no iron deficiency and the gene expression of Fe-related proteins was evaluated in the liver and intestine of non-anemic animals. However, the PB group presented lower hemoglobin gain than other groups, and can be associated with the lower gene expression of Fe-related proteins, which can impair the iron absorption and its incorporation in hemoglobin.

The lower expression of these proteins may also be associated with a lower antioxidant activity observed in this group, since oxidative stress can increase production of hepcidin, which can reduce gene expression of DMT-1 and ferroportin [45,47,48].

The molar ratio of zinc: iron diets also did not differ (0.18:1 in average). Tripathi *et al*., (2012) [49] found in an *in vitro* study that a molar ratio of zinc: iron of 0.5 did not reduce the bioavailability of iron. However, the PB group showed lower bioavailability of iron compared to the other groups, which may also be associated with reduced gene expression of DMT-1 observed in this group, since the iron and zinc compete for absorption sites [50].

Conversely, Tako *et al.* (2013) [14] observed higher HG and HRE in birds consuming common beans biofortified with iron compared to the regular common bean. Furthermore, they observed lower gene expression of DMT-1, ferroportin and DcytB.

When Pontal bean was associated with rice (PB + R) the bioavailability of iron increased, as demonstrated by the indices of HRE and Relative Biological Value of HRE (RBV-HRE). This result may be related to the fact that this combination provided an increased amount of sulfur amino acids such as cysteine, which has been reported to promote the bioavailability of iron [4,51]. In addition, this group increased the gene expression of ferritin and transferrin, contributing to increased iron bioavailability.

The PB + R group also increased the gene expression of ferritin and transferrin. This may have occurred due to low iron intake during the repletion phase, which may have increased the gene expression of transferrin in the liver tissue, in order to compensate the reduced supply of this mineral. Increased gene expression of transferrin, in turn, increased iron transport to the liver leading to increased gene expression of ferritin in order to store the transported iron. The increase in iron storage in the liver resulted in reduced availability for hematopoiesis, which was evidenced by the reduced HG displayed by this group.

The hephaestin mRNA was more expressed in the group fed with rice, beans and high pro-vitamin A carotenoid content crops (PB + R + P and PB + R + SP), suggesting an increase in iron transport into the bloodstream. However, this did not show a significant difference in HG and HRE in the other groups, except for the PB group.

In addition, in the present study despite the high concentration of phenolic compounds in food, it probably did not affect the iron bioavailability. However, the polyphenols were evaluated by a method that does not measure specific polyphenols and/or flavonoids compounds. In contrast, some studies have reported a negative effect of several polyphenols on the iron bioavailability [45,46]. The concentration of phenolic compounds in pumpkin and sweet potato may also have contributed to the increased antioxidant activity of food combinations.

The control group showed plasma TAC similar to the experimental groups, except for the PB group. It can be explained by the presence of ferrous sulfate, used as a regular iron source in the control diet. This form of iron has high bioavailability, resulting in increased iron absorption. The increase of iron in the liver tissue may increase the production of reactive oxygen species resulting in an increased activity of superoxide dismutase (SOD) [52], which may be responsible for this increase in the plasma TAC. In addition, the TAC has been evaluated in the plasma of animals in a 12 h fast and some studies have found no difference in TAC animals in this state [53,54].

The group PB + R also presented TAC comparable to the groups supplemented with pro-vitamin A carotenoids. This group, despite not having consumed high pro-vitamin A carotenoid content crops, showed high gene expression of ferritin and transferrin, binding iron proteins that are also part of an antioxidant defense system [55]. According to Cornelis *et al.*, (2012) [56] control of iron absorption prevents the accumulation of toxic levels of iron which can promote the production of reactive oxygen species (ROS), suggesting a link between the regulation of iron homeostasis and resistance to oxidative stress mechanisms.

## 5. Conclusions

The combination of rice and common beans, targets for biofortification, together with high pro-vitamin A carotenoid content crops (pumpkin and sweet potato) increased plasma antioxidant capacity and gene expression of proteins involved in iron metabolism, favoring an increase in iron bioavailability.
